# Is There Evidence of Cost Benefits of Electronic Medical Records, Standards, or Interoperability in Hospital Information Systems? Overview of Systematic Reviews

**DOI:** 10.2196/medinform.7400

**Published:** 2017-08-29

**Authors:** Zilma Silveira Nogueira Reis, Thais Abreu Maia, Milena Soriano Marcolino, Francisco Becerra-Posada, David Novillo-Ortiz, Antonio Luiz Pinho Ribeiro

**Affiliations:** ^1^ Informatics Center in Health Obstetrics and Gynecology Department Universidade Federal de Minas Gerais Belo Horizonte, Minas Gerais Brazil; ^2^ State Health Secretariat of Minas Gerais Planning Advisory Belo Horizonte, Minas Gerais Brazil; ^3^ Faculty of Medicine Internal Medicine Department Universidade Federal de Minas Gerais Belo Horizonte, Minas Gerais Brazil; ^4^ Pan American Health Organization Washington, DC, DC United States; ^5^ Medical School, Telehealth Center, Hospital das Clínicas Internal Medicine Department Universidade Federal de Minas Gerais Belo Horizonte, Minas Gerais Brazil

**Keywords:** electronic medical records, standards, medical information exchange, health information exchange, cost, benefits and costs

## Abstract

**Background:**

Electronic health (eHealth) interventions may improve the quality of care by providing timely, accessible information about one patient or an entire population. Electronic patient care information forms the nucleus of computerized health information systems. However, interoperability among systems depends on the adoption of information standards. Additionally, investing in technology systems requires cost-effectiveness studies to ensure the sustainability of processes for stakeholders.

**Objective:**

The objective of this study was to assess cost-effectiveness of the use of electronically available inpatient data systems, health information exchange, or standards to support interoperability among systems.

**Methods:**

An overview of systematic reviews was conducted, assessing the MEDLINE, Cochrane Library, LILACS, and IEEE Library databases to identify relevant studies published through February 2016. The search was supplemented by citations from the selected papers. The primary outcome sought the cost-effectiveness, and the secondary outcome was the impact on quality of care. Independent reviewers selected studies, and disagreement was resolved by consensus. The quality of the included studies was evaluated using a measurement tool to assess systematic reviews (AMSTAR).

**Results:**

The primary search identified 286 papers, and two papers were manually included. A total of 211 were systematic reviews. From the 20 studies that were selected after screening the title and abstract, 14 were deemed ineligible, and six met the inclusion criteria. The interventions did not show a measurable effect on cost-effectiveness. Despite the limited number of studies, the heterogeneity of electronic systems reported, and the types of intervention in hospital routines, it was possible to identify some preliminary benefits in quality of care. Hospital information systems, along with information sharing, had the potential to improve clinical practice by reducing staff errors or incidents, improving automated harm detection, monitoring infections more effectively, and enhancing the continuity of care during physician handoffs.

**Conclusions:**

This review identified some benefits in the quality of care but did not provide evidence that the implementation of eHealth interventions had a measurable impact on cost-effectiveness in hospital settings. However, further evidence is needed to infer the impact of standards adoption or interoperability in cost benefits of health care; this in turn requires further research.

## Introduction

Information technology (IT) applied to health care, or electronic health (eHealth) [1], ostensibly offers numerous benefits to the quality of health information, particularly in its recording, retrieval, and use. Patients can benefit directly from safe and accessible electronic clinical information for better decision making [2]. However, demographics and patient data are highly fragmented and distributed across multiple unintegrated systems [3]. Comprehensive and consistent health care, leading to effective use of services, requires the computerization of health data for more efficient communication. To achieve this, standardized information channels are needed to make syntactic interoperability possible among electronic records systems. Semantic interoperability is necessary to guarantee the consistency of information, as health information models require adopting standards to support communication [2]. Even if the standardization of electronic health records (EHRs) in eHealth systems is accomplished, health data sharing will continue to be a global challenge. Few publications exist concerning the impact of medical records and interoperability among health systems in cost and benefits of patient care.

Improvements in health and economic indicators are relevant metrics to justify IT investments. Indeed, planning and investing in IT is necessary for the efficient use of information that not only advances health care but also holds financial, social, cultural, and ethical benefits. Comparative cost-effectiveness studies guide agencies and institutions in choosing the best option for desired clinical outcomes and costs, which is the key to ensuring the sustainability of government health systems and their welfare programs [3,4].

This review analyzes systematic reviews addressing the cost benefit and effectiveness of electronic medical records (EMR), standards adoption, or interoperability to discuss the benefits, drawbacks, and lessons learned from the implementation of actions related to eHealth and serves as a reference for government representatives and stakeholders. The assessment of the involvement of government and private health institutions in the implementation and maintenance of eHealth interventions that were tested and valuated worldwide is also of interest. The study was directed by 2 questions: What evidence exists regarding the impact of computerizing applications, standards, health information exchange, or interoperability to support the quality of care or patient outcomes in hospital settings? What critical cost-benefit evidence is published to provide a clear understanding of the value of eHealth implementations?

## Methods

### Basic Concepts and International Standards

#### On eHealth

The terms used here to describe eHealth technologies are available in Multimedia Appendix 1. Despite different meanings, some papers use the terms electronic medical record (EMR) and electronic health record (EHR) synonymously. A relevant dissimilarity exists between health information exchange for systems integration and interoperability. The former refers to organizational framework for the dissemination of electronic health care information or clinical data across health-related institutions and systems to enhance patient care [5]. The latter relates to the the ability or capability of two or more systems to exchange information and use the exchanged information, which may support a longitudinal record widely available across institutions and over life spans [6]. Additionally, in a more specific context, “interoperability means the ability of health information systems to work together within and across organizational boundaries in order to advance the effective delivery of health care for individuals and communities” [7].

It is also important to emphasize that interoperability is usually divided into (1) syntactic interoperability: the capability of two or more systems to communicate and exchange data through specified data formats and communication protocols, and (2) semantic interoperability: the ability for data shared by systems to be understood at the level of fully defined domain concepts [8].

Worldwide coordinated efforts resulted in the development of standards to define an EHR as one or more repositories of actionable information by computers. The European Committee for Standardization (CEN), health level seven (HL7), International Organization for Standardization (ISO), and openEHR Foundation are nonprofit organizations dedicated to providing frameworks and standards. Terminologies, EHR specifications, and information models are proposed by these international standards organizations that support the exchange, integration, interoperability, and retrieval of electronic health information [6].

To better represent the meaning of standards in the primary selected systematic reviews, we adopted the generic definition for the term as: “A document adopted by consensus by a recognized entity, that provides rules, guidelines and/or features for common use, in order to obtain an optimal level of performance in a given context…” [9].

#### On Economic Analysis

Economic analysis supports health care policy and organizational decision making. However, it encounters some difficulties with eHealth systems, which are as follows: constantly changing technology, inconsistent study design to manage inadequate sample sizes, the inappropriateness of conventional techniques of economic evaluation, and the problem of placing value on health and nonhealth outcomes [10]. Consequently, five methods have been used to calculate the cost-effectiveness of traditional and eHealth interventions: cost-minimization analysis, cost-benefit analysis, cost-effectiveness analysis, cost-utility analysis, and cost-consequence analysis [11].

### Data Sources and Search Strategy

This review of systematic reviews has been conducted in accordance with the preferred reporting items for systematic reviews and meta-analyses (PRISMA) statement [12] and the recommended methodological considerations when using existing systematic review as described by Whitlock et al [13].

On February 22, 2016, electronic searches were conducted on the MEDLINE, Cochrane Library, LILACS, and IEEE Library databases. To identify the EHR concept, standards for interoperability, and health information and its cost benefits, the search strategy was:

((“Electronic health records”[MeSH Terms] OR “Health Information Exchange”[MeSH Terms] OR (“Health Information Management”[MeSH Terms] OR (“Medical Informatics” [MeSH Terms]) AND (“Interoperability” OR “Standard of Information”)) AND (“Cost-Benefit Analysis”[MeSh] OR “Evaluation Studies”[Publication Type] OR “Program Evaluation”[MeSh] OR impact or effectiveness)

The search was limited by language of publication (English, Spanish, French, Italian, and Portuguese), studies in humans, type of study (systematic reviews and meta-analyses), and year of publication (since 2005). Two systematic reviews that satisfied the criteria were identified manually. To better define certain eHealth technology descriptions, additional sources of evidence were considered.

### Study Selection

The inclusion criteria were as follows:

1. Primary impact: EMR, standards, or interoperability on cost-benefit, or
2. Secondary impact: EMR, standards, or interoperability on quality of care (clinical outcomes), and
3. Real-life reviews about interventions in in-hospital settings.

Studies in primary or secondary care scenarios, studies without the primary or secondary impact of eHealth actions, and duplications were excluded. Titles and abstracts of retrieved papers were independently screened and evaluated by 2 investigators (ZSNR and TAM). Abstracts providing insufficient information were retrieved for independent, full-text evaluation by 2 investigators to determine study eligibility. Disagreements were resolved by consensus. Additional publications were identified using the reference lists of selected manuscripts.

### Data Extraction and Quality Assessment

ZSNR prepared electronic data with paper contents abstracted using StArt software (Systematic Review System) to organize the analysis [14]. The data extraction of full-text analysis included the following: study design, number of studies evaluated, objectives, type of interventions/clinical data sources, eHealth interventions and terminology, interface/health information exchanges, duration of follow-up, cost-effectiveness, impact on quality of care, main results control group, potential bias, limitations, and lessons learned. The results were summarized into two subgroups according to the modality of intervention:

- Subgroup 1: eHealth systems implementation without health information exchange
- Subgroup 2: eHealth systems with health information exchange functionalities

The methodological quality assessment was based on the AMSTAR (a measurement tool to assess systematic reviews) checklist [15].

## Results

**Figure 1:**
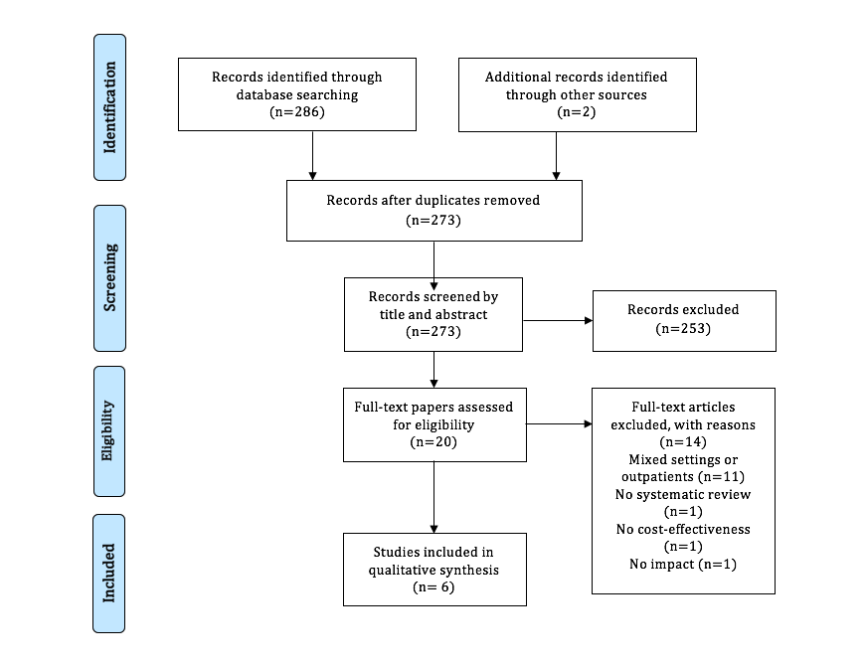
Flow of information through the different phases of the systematic review.

A total of 288 papers were identified during the initial research phase, which decreased to 273 after removing 15 duplicates. After applying our criteria, only six systematic reviews were included in the final analysis and data-abstraction phase. The review process is represented in Figure 1, according PRISMA Statement [14].

The primary cause for excluding the 20 studies was mixed or outpatient settings for eHealth interventions (11 papers of 14 excluded, 79%). The Pan American Health Organization (PAHO) conducted a review of the implementation and effective use of standards to achieve interoperability in Latin American and Caribbean countries but without direct or indirect outcomes analysis [6]. Multimedia Appendix 2 presents a detailed summary of the 14 full-text excluded systematic reviews.

### Characteristics and Quality of the Selected Studies

Evidence of the cost-effectiveness of eHealth interventions that met the criteria was identified. Only one systematic review of the six performed a meta-analysis [16]. The quality assessment of the included studies followed AMSTAR (a measurement tool to assess systematic reviews) methodology and resulted in wide variability of the quality score. Two studies were classified with a moderate rating of quality with 5 positive points among 11 items [16,17], whereas other reports neglected many AMSTAR criteria [18-21]. Table 1 summarizes the quality assessment ratings, the study design, and the funding or support of the six included systematic reviews.

**Table 1:** Quality assessment ratings and characteristics of the six included systematic reviews.

Study	AMSTAR^a^ score				Funding or support	Study design	Number of studies evaluated	Control group (most frequent)	Meta- analysis
	Y^b^	N^c^	CA^d^	N/A^e^					
Thompson et al 2015 [16]	4	7	0	0	Y	RCT^f^, pre-post studies, descriptive studies	45 total/Meta-analysis: 26	Pre-post implementation (paper vs system)	Y
Cheung et al 2015 [17]	5	4	0	2	NC^g^	RCT, quasi-experimental studies, descriptive studies	18	Pre-post implementation	N
de Bruin et al 2014 [20]	2	7	0	2	NC	Quasi-experimental	26	True infection detection by infection control experts	N
Mapp et al 2013 [21]	1	7	1	2	NC	Observational, Pilot studies	9	No control	N
Li et al 2013 [18]	4	5	0	2	NC	RCT, quasi-experimental studies	6	Patient not reported in written notes or before system	N
Govindan et al 2010 [19]	5	4	0	2	Y	Observational: accuracy of the automated method with a gold standard method	43	Standard chart review	N

^a^AMSTAR: a measurement tool to assess systematic reviews.

^b^Y: yes.

^c^N: no.

^d^CA: cannot answer.

^e^N/A: not applicable.

^f^RCT: randomized controlled trial.

^g^NC: not commissioned.

Table 2 summarizes the objective, the type of intervention/clinical data sources, eHealth intervention and terminology, interface/health information exchange, and duration of follow-up of the six included systematic reviews.

### Summarized Outcomes

Among the included systematic reviews, only one was classified as showing an effect on eHealth implementation without electronic health information exchange (Subgroup 1), and the other five were ranked as showing effects of systems implementation with incorporated health information exchange among other electronic data sources (Subgroup 2).

#### Subgroup 1

Considering eHealth systems implementation without health information exchange, the review of Thompson et al [16] reported a parallel to advances in digital technology and how different forms of eHealth systems have been developed and implemented (Table 3).

##### Types and Functions of Technology Systems

The selected review stated a mix of electronic interventions: EHR, EMR, computerized decision support systems (CDSS), computerized provider order-entry (CPOE) and surveillance systems used by physicians, nurses, allied health professionals, and managers of health services evaluating evidence from pre-and postsystems implementation. The analysis synthesized 46 publications about systems for diagnosis, treatment, and clinical monitoring. The study included a meta-analysis extracted from 26 publications to evaluate the effects of different types of systems regarding health IT in the inpatient of intensive care unit (ICU) setting on mortality, length of stay (LOS), and cost.

##### Effects on Quality or Efficiency of Care

Not enough evidence showed that electronic interventions can improve quality and safety of health care. The goals for secondary outcomes were the effects of health IT in the inpatient and ICU on mortality or LOS. The quality of included studies and interventions varied significantly, which was highlighted as the major limitation. Despite this, the surveillance systems had a pooled odd ratio (OR) of 0.85 (95% CI 0.76-0.94) with moderate heterogeneity, I^2^ of 59%.

##### Effects on Costs

Costs were unable to be evaluated quantitatively because the primary studies presented mixed and inconclusive results, leaving us unable to draw a definitive conclusion about cost-effectiveness. The analysis of costs was more limited than the evidence on quality and efficiency.

#### Subgroup 2

EHR implementation with health information exchange is a recent worldwide trend in hospital settings. A summary of the results of the systematic reviews included in subgroup 2 is presented in Table 4.

**Table 2:** Descriptive summary of the systematic reviews included in electronic medical records (EMRs)/Interoperability review.

Study	Objective	Type of intervention/ Clinical data sources	eHealth intervention and terminology	Interface/health information exchange	Duration of follow-up
Thompson et al 2015 [16]	To evaluate effects of health IT^a^ in the inpatient and ICU^b^ on mortality, LOS^c^,, and cost	Multiple health IT interventions on diagnosis, treatment, monitoring, cost reduction/No reference	EHR^d^, EMR^e^, CDSS^f^,CPOE^g^, Surveillance system	No reference	No reference
Cheung et al 2015 [17]	To evaluate the effects of an information system integrated to PDMS^h^ on organizational and clinical outcomes, in ICU^i^/Operating room	Integrating bedside equipment to an information system/vital signs, patient monitor, ventilator, anesthesia machine, dialysis machine, IV pump, lab values, hospital information system, admission, discharge and transfer	CDSS, PDMS, health information exchange	PDMS to an information system/no mention about direction of data exchange	1 day to 1 week;11 months to 4 years
de Bruin et al 2014 [20]	To evaluate recent trends in use of electronically available patient data by electronic surveillance systems for HAIs^j^ and identify consequences for system effectiveness	HAIs that utilize EHR available in hospitals to surveillance the HAIs/Medico-administrative data procedures or discharge reports, free text reports, biochemistry, microbiology, and radiology laboratory test results, pharmacy dispensing records, radiology free-text records, vital signs, electronic discharge summary	Automated detection by HAI systems: EHR, health information exchange, using ICD^k^-9, ICD-10, discharge coding, ATC^l^ code	EHR to HAI systems/no mention about direction of data exchange	No reference
Mapp et al 2013 [21]	To examine early warning scoring systems and their effectiveness in predicting a patient's potential for deterioration and considers whether these scoring systems prevent unplanned ICU admissions and/or death	Instruments and clinical support systems available to assist health care personnel in recognizing early clinical deterioration/Vital signs, SpO_2_^m^, LOC^n^, UOP^o^, nurse/family concerns, complaints, lab values	EMR, CDSS, health information exchange based on SBAR^p^ communication	Early warning scoring systems that interface with EMRs and are supplemented with decision aides (algorithms) and clinical support systems/no mention about direction of data exchange	Seven studies: 3 to15 months/two studies: over 24 months to 8 years
Li et al 2013 [18]	To evaluate the impact of the CHTs^q^ on the quality of physician handoff, patient care, and physician work efficiency	Decision support/training, emergency referrals, supervision, alerts and reminders, client education, data collection, medicine dosing/Patient demographics, medications, diagnosis, problem lists, comment line, vital signs, to-do list, LOS, free daily notes, lab values	CHTs, EMR, CDSS, health information exchange. Allergy Code	Clinical information exchange using CHTs for physician handoff for hospitalized patients CHTs/mixed (no interface, unidirectional or bidirectional interface exchange)	1 to 6 months
Govindan et al 2010 [19]	To identify, describe, and evaluate the effectiveness of automated inpatient harm-detection methods	Automated harm detection on EMR. Gold standard: chart review	Automated detection by surveillance systems: EMR, health information exchange, using ICD-9, procedure codes, billing codes	Automated harm detection on EMR, using field-defined systems, natural language-processing/Unidirectional retrospective	No reference

^a^IT: information technology.

^b^ICU: intensive care unit.

^c^LOS: length of stay.

^d^EHR: electronic health record.

^e^EMR: electronic medical record.

^f^CDSS: computerized decision support systems.

^g^CPOE: computerized provider order-entry.

^h^PDMS: Patient data management system.

^i^ICU: intensive care unit.

^j^HAIs: health care–associated infections systems.

^k^ICD: international classification of disease.

^i^ATC: anatomical therapeutic chemical.

^m^SpO_2_: oxygen saturation.

^n^LOC: level of consciousness.

^o^UOP: urine output.

^p^SBAR: situation, background, assessment, recommendation.

^q^CHTs: computerized physician handoff tools.

**Table 3:** Descriptive summary of the results of systematic reviews included in electronic medical records(EMRs)/Interoperability review. Subgroup 1: electronic health (eHealth) systems implementation without health information exchange.

Study	Primary impact: Cost-effectiveness	Secondary impact: Quality of care/ Clinical outcome	Main results	Potential bias	Lessons
Thompson et al 2015 [16]	Mixed and inconclusive	Mortality: overall CPOE^a^ systems did not show a significant effect (OR^b^: 0.91, 95% CI 0.75-1.10; I^2c^ 66%), nor EHR^d^ alone (OR: 0.96, 95% CI 0.77-1.19). CDSS^e^(OR 0.96, 95% CI 0.77-1.19). The surveillance systems had a pooled OR of 0.85 (95% CI 0.76-0.94) with moderate heterogeneity, I^2^59% LOS: CPOE trended toward a reduction in LOS (mean decrease, 0.67 days, 95% CI –2.07 to 0.73), though with significant heterogeneity (I^2^82%). Neither CDSS nor surveillance systems trended toward changes in hospital LOS, and the net-pooled effect was not significant.	Electronic interventions were not shown to have a substantial effect on mortality, LOS^f^, or cost.	Selection, measurement	There is not enough evidence to confidently state that electronic interventions have the ability to achieve the goal of improving quality and safety.

^a^CPOE: computerized provider order-entry.

^b^OR: odds ratio.

^c^I^2^: measure of heterogeneity.

^d^EHR: electronic health record.

^e^CDSS: computerized decision support systems.

^f^LOS: length of stay.

**Table 4:** Descriptive summary of the results of systematic reviews included in the electronic health record (EHR)/Interoperability review. Subgroup 2: electronic health (eHealth) systems implementation with information exchange.

Study	Primary impact: Cost-effectiveness	Secondary impact: Quality of care/ Clinical outcome	Main results	Potential bias	Lessons
Cheung et al 2015 [17]	Not evaluated^a^	PDMS^b^reduced charting time, increased time spent on direct patient care and reduced the occurrence of errors (medication errors, intravenous and ventilation incidents). The effect on documentation was mixed. Improvement in clinical outcomes when PDMS was integrated with a CDSS^c^, but scarce literature is available.	The effect on documentation was mixed. Qualitative analysis showed a significant decrease in time spent on documentation. Clinical outcomes: inconclusive.	Selection, measurement	Improvement in clinical outcomes when PDMS was integrated with a CDSS, but there is scarce literature available. Organizational advantages included improved accuracy, legibility, data accessibility, and decision support. Such integration may improve clinical outcomes, although further studies are required for validation.
de Bruin et al 2014 [20]	Not evaluated^a^	Electronic surveillance achieves equal or better sensitivity than manual surveillance. Several studies also reported time savings of 60% to 99.9% or a reduction in chart reviews of 40% to 90.5%.	Driven by the increased availability of electronic patient data, electronic HAIs^d^surveillance systems use more data, making systems more sensitive yet less specific but also allow systems to be tailored to the needs of health care institutes’ surveillance programs.	Selection	HAIs detection systems use increasingly more EHR^e^and patient data as more data sources become available. Thus, systems tend to become more sensitive and less specific.
Mapp et al 2013 [21]	Not evaluated^a^	An increase occurred in the number of rapid response calls by nursing staff, a decrease in unplanned ICU^f^admissions, and a decrease in hospital mortality.	Improvement in clinical outcomes when using early warning scoring systems.	Selection	Early warning scoring systems can be more effective with the integration of algorithms and clinical support systems.
Li et al 2013 [18]	Not evaluated^a^	Impact on physician work efficiency (self-reported time spent on handing copying patient information; 50%) and proportionally more time to see patients. Time on each patient during rounding decreased by1.5 min. Impact on quality on physician handoff: completeness and consistency of the handoff document has improved.	Completeness and consistency of the handoff document has improved. Accuracy of information about patients during physician handoff.	Selection, measurement	CHTs^g^could potentially enhance work efficiency and continuity of care during physician handoff, but the role in improving quality is less clear. The information available was often not sufficient to help on-call physicians make patient care decisions.
Govindan et al 2010 [19]	Not evaluated^a^	Sensitivities of different methods ranged from 0.10 to 0.94, specificity from 0.10 to 0.94, PPV^h^from 0.03 to 0.84, and NPV^i^from 0.70 to 0.96. The field-defined methods of automated harm detection will prove superior to natural language processing, particularly if information about harm is accurately documented.	Automated harm detection has the potential to positively influence clinical practice.	Selection, measurement	Automated harm detection has the potential to positively influence clinical practice. Another potential benefit is the reduction of person-hour required to harm surveillance.

^a^Not evaluated in the selected study.

^b^PDMS: Patient data management system.

^c^CDSS: computerized decision support systems.

^d^HAIs: health care–associated infections systems.

^e^EHR: electronic health record.

^f^ICU: intensive care unit.

^g^CHTs: computerized physician handoff tools

^h^PPV: positive predictive value.

^i^NPV: negative predictive value.

##### Types and Functions of Technology Systems

Most of the reviews use ICUs as settings for eHealth intervention analysis. However, the objectives of interventions were quite heterogeneous. Two studies reported the effect of surveillance systems on harm detection [19] and health care–associated infections [20]. Bedside data integration in an information system [17], continuity of care using physician handoff tools [18], and prediction of death or unexpected ICU admission [21] were the proposals of the other reviews. Regarding application users, two studies focused on patient outcome results for health care managers [19,20]. Some focused directly on health care professionals to improve clinical practice [18,19,21]. On the direction of electronic health information exchange, one review described it as unidirectional [19], three did not clarify whether the exchange was bidirectional [17,20,21], and one summarized mixed studies including systems without interfaces [18]. None mentioned interoperability among electronic health systems. Regarding standards for the exchange of clinical data, four studies reported the use of terminologies such as International Classification of Disease (ICD) and anatomical therapeutic chemical (ATC) code [18-21].

##### Effects on Quality or Efficiency of Care

Among reviews focused on improving clinical practice, inconclusive results in direct patient care were reported by Cheung et al [17]. Mapp et al [21] highlighted an increase in nursing staff efficiency regarding rapid calls response, a decrease in unplanned ICU admissions, and hospital mortality. Li et al [18] presented a positive impact on continuity of inpatient care. With regard to indirect results on patient care, two studies highlighted the improvement of health data quality in terms of accuracy, legibility, completeness, and consistency of documents [17,18]. The other reviews focused on electronic surveillance. The results showed that systems tend to become more sensitive and less specific than manual monitoring to detect infection [20]. With respect to inpatient harm detection, the automated systems allowed rapid scanning of a vast number of patient records with minimal effort and may identify events as they occur in real time [19]. Most automated surveillance systems were retrospective, but some real-time surveillance alerts that informed physicians and pharmacists of adverse events were reported [19].

##### Effects on Costs

None of the reviews evaluated effects of eHealth interventions on costs.

## Discussion

### Principal Findings

This study found preliminary benefits in the use of electronically available inpatient data systems on the quality of care. Despite the limited number of studies that met the eligibility criteria, the heterogeneity of electronic systems reported, and different interventions on hospital routines, the identification of preliminary secondary benefits on patient mortality was possible [16]. eHealth systems with information exchange functionalities also showed potential impact on quality of care or patient outcomes. From five studies, one had inconclusive results on direct patient care [17] and four presented partial effects, as nursing staff efficiency led to a faster call response, a decrease in unplanned ICU admissions and hospital mortality [18], improvement of health data quality [17,18], and more efficient surveillance programs inside hospitals [19,20]. It is expected that the systems able to share health information would improve care at the time and point of attention, especially the surveillance systems and those that use common terminologies and vocabularies to support consistency in information collection [6,19,20,22].

However, no substantial review regarding the impact of electronic interventions on cost-effectiveness was identified. Among the six analyses included, only Thompson et al reported that some preliminary studies have identified decreases in cost, but the heterogeneity and the absence of information of follow-up impaired a proper analysis of cost-effectiveness [16]. Immediate cost savings are not anticipated for organizations when choosing to adopt eHealth strategies because the high cost of implementation limits the transition from paper-based to electronic systems and represents a significant challenge to their widespread adoption [23]. Regardless, medium and long-term positive results are expected, and the World Health Organization (WHO) recognized overall eHealth as cost- effective and secure [24]. Potential indirect cost saving was mentioned as a secondary outcome in three studies, with the reduction of person-hours harming surveillance and the increase in time spent on direct patient care [17,18,24].

Unfortunately, no study about interoperability, in the sense of syntactic and semantic meaning, on cost benefit was identified. Importantly, none of the studies in this review properly defined EHR concept as a longitudinal health record with entries by health care practitioners in multiple sites of care or mentioned interoperability applications among electronic systems. However, taking the antecedent step toward full interoperability, an effective information sharing between stakeholders and systems can be attained through the use of standards [6]. Standards adoption for the exchange of clinical data was mentioned in four studies [18-21], mostly terminologies adoptions, but the potential impact of such tools on continuity of care or costs remains an open question that needs investigation. Although within the limits of hospital systems, the analysis confirmed the potential to positively impact physician practice organizations, as previously reported [23]. Further longitudinal research is needed to determine the actual impact of eHealth adoption on health care costs and clinical outcomes.

### Limitations

The current results should be interpreted as a whole with the study limitations. Only four major databases were searched and gray literature sources were not evaluated. Additionally, the limitation to English, Spanish, French, Italian, and Portuguese languages prevented the capture of all relevant studies. Furthermore, the quality of included studies was poor, and they varied regarding the type of eHealth interventions, follow-up time, and goals. This systematic review summarized primary and secondary outcomes from different classes of intervention from which to draw results, analysis, and conclusions. Due to the variation in scenarios and lack of numeric goals, a meta-analysis was considered inappropriate.

### Conclusions and Lessons Learned

This review identified some benefits on the quality of care but did not provide evidence that the eHealth interventions had a measurable impact on cost-effectiveness, mortality, or LOS in hospital settings. Preliminary evidence indicates that the use of eHealth interventions with information exchange may improve clinical process outcomes. The absence of studies precludes the assessment of impact of interoperability on benefits of health care or cost, and this aspect needs further research. Technological barriers might influence eHealth solutions implementation and data exchange for systems integration or interoperable interfaces. There are also issues with the lack of standardization of most aspects of health information and misuse of terms in the scientific publications. Authors should be explicit when they are using interfacing syntactic interoperability or semantic interoperability to reduce the confusion with different health information exchange possibilities. Further research with long-term follow-up is needed to determine the actual impact of eHealth adoption on health care costs to demonstrate (1) value for money (including clinical impacts) and (2) the clinical impact of semantic and synthetic interoperability.

## Appendix

Multimedia Appendix 1 eHealth concepts and definitions.

Multimedia Appendix 2 Descriptive summary of the 14 full-text excluded systematic reviews.

## Abbreviations

AMSTAR: a measurement tool to assess systematic reviews
ATC: anatomical therapeutic chemical
CDSS: computerized decision support systems
CEN: European Committee for Standardization
CHTs: computerized physician handoff tools
CPOE: computerized provider order-entry
CPR: computer-based patient record
EHR: electronic health record
EMR: electronic medical record
HAIs: health care–associated infections surveillance systems
HL7: Health level seven
I2: measure of heterogeneity
ICD: International Classification of Disease
ICU: Intensive Care Unit
ISO: International Organization for Standardization
IT: information technology
LOC: level of consciousness
LOS: length of stay
NPV: negative predictive value
OR: odds ratio
PAHO: Pan American Health Organization
PDMS: patient data management system
PPV: positive predictive value
PRISMA: preferred reporting items for systematic reviews and meta-analyses
SpO2: oxygen saturation
SBAR: situation, background, assessment, recommendation
UOP: urine output
WHO: World Health Organization
