# Supplementary material for: Is There Evidence of Cost Benefits of Electronic Medical Records, Standards, or Interoperability in Hospital Information Systems? Overview of Systematic Reviews
Source: JMIR Med Inform. 2017 Aug 29;5(3):e26. doi: 10.2196/medinform.7400 (PMC5596299; doi:10.2196/medinform.7400)
Supplement: Multimedia Appendix 1 [file medinform_v5i3e26_app1.pdf]

Multimedia Appendix 1. eHealth concepts and definitions

Concept	Definition	Reference
Automated health records	“A collection of computer-stored images of traditional health record documents. Typically, these documents are scanned into a computer and the images are stored on.”	[2]
Computer-based patient record (CPR)	“A collection of health information for one patient linked by a patient identifier. The CPR could include as little as a single episode of care for a patient or healthcare information over an extended period of time.”	[2]
Computerized decision support systems (CDSSs)	“Any electronic system designed to aid directly in clinical decision making, in which characteristics of individual patients are used to generate patient-specific assessments or recommendations that are then presented to clinicians for consideration”. So, CDSSs match individual patient characteristics to a computerized knowledge base, and software algorithms generate patient-specific recommendations. Examples: alerts, reminders, order sets, drug-dosage calculations, and care-summary dashboards that provide performance feedback on quality indicators or benchmarks.	[25]
eHealth	Mitchel (1999) defined eHealth as “the combined use in the health sector of electronic communication and information technology (digital data transmitted, stored and retrieved electronically) for clinical, educational and administrative purposes, both at the local site and at a distance” [26].<br>Eysenbach (2001) defined eHealth as “an emerging field in the intersection of medical informatics, public health and business, referring to health services and information delivered or enhanced through the Internet and related technologies. In a broader sense, the term characterizes not only a technical development, but also a state-of-mind, a way of thinking, an attitude, and a commitment for networked, global thinking, to improve health care locally, regionally, and worldwide by using information and communication technology” [27].	[26, 27]
Electronic health records (EHR)	The term electronic health record has variations in definitions and extent of coverage in different countries/institutions. It may be a longitudinal record widely available across a number of institutions or just a limited automated system only available within a specific unit or department.<br>In a broad sense, it may be defined as a longitudinal health record with entries by healthcare practitioners in multiple sites where care is provided:<br>• Contains all personal health information belonging to an individual;<br>• Is entered and accessed electronically by [illegible]	[2]

References

1. World Health Organization. Apps.WHO. 2006. Electronic health records: manual for developing countries [accessed 2017-07-13]http://apps.who.int/iris/handle/10665/207504[Webcite Cache]
6. Pan American Health Organization. Webcitation. Pan American Health Organization; 2016. eHealth in Latin Americathe Caribbean: interoperability standards review [accessed 2017-07-14]http://www.webcitation.org/6rwrfeqBJ[Webcite Cache]
7. Webcitation. 2005. Interoperability Definition and Background [accessed 2017-07-14]http://www.webcitation.org/6rKvIpuvO[Webcite Cache]
9. International Organization for Standarization - ISO. Webcitation. Standardization and related activities -- General vocabulary [accessed 2017-07-14]http://www.webcitation.org/6rKvaBnAJ[Webcite Cache]
17. Cheung A, van Velden FH, Lagerburg V, Minderman N. The organizational and clinical impact of integrating bedside equipment to an information system: a systematic literature review of patient data management systems (PDMS). Int J Med Inform 2015 Mar; 84(3):155-65[Medline][CrossRef].
25. Kawamoto K, Houlihan C, Balas E, Lobach D. Improving clinical practice using clinical decision support systems: a systematic review of trials to identify features critical to success. Br Med J 2005; 330(7494):765[CrossRef]
26. Mitchell J. From Telehealth to E-health: The Unstoppable Rise of E-health. Canberra, NSW: Commonwealth Department of Communications, Information Technology and the Arts; 1999. 064275036
27. Eysenbach G. What is e-health?. J Med Internet Res 2001 Jun; 3(2):E20[FREE Full text][Medline][CrossRef]
28. Pan American Health Organization. PAHO. Washington, DC: PAHO HQ Library Cataloguing-in-Publication; 2011. Epidemiological Surveillance of Healthcare Associated Infections [accessed 2017-07-14]http://new.paho.org/hq/dmdocuments/2011/ENG_Modulo_I_final.pdf[Webcite Cache]
29. Main C, Moxham T, Wyatt J, Kay J, Anderson R, Stein K. Computerised decision support systems in order communication for diagnostic, screening or monitoring test ordering: systematic reviews of the effects and cost-effectiveness of systems. Health Technol Assess 2017 Jul 14; 14(48):1-227[FREE Full text][Medline][CrossRef]
