# Supplementary material for: Is There Evidence of Cost Benefits of Electronic Medical Records, Standards, or Interoperability in Hospital Information Systems? Overview of Systematic Reviews
Source: JMIR Med Inform. 2017 Aug 29;5(3):e26. doi: 10.2196/medinform.7400 (PMC5596299; doi:10.2196/medinform.7400)
Supplement: Multimedia Appendix 2 [file medinform_v5i3e26_app2.pdf]

Multimedia Appendix 2. Descriptive summary of the fourteen full-text excluded systematic reviews

Study	Impact of eHealth actions analysis	Theoretical scenario	Setting: includes outpatient interventions	Review of Systematic Review
Chaudhry et al., 2006 [30]	Y[a]	N[b]	Y[a]	Y[a]
Jamal et al., 2009 [31]	Y[a]	N[b]	Y[a]	Y[a]
Wollersheim et al., 2009 [32]	Y[a]	Y[a]	...	N[b]
Main et al., 2010 [29]	Y[a]	N[b]	Y[a]	Y[a]
Police et al., 2010 [23]	Y[a]	N[b]	Y[a]	Y[a]
Chan et al., 2012 [33]	N[b]	N[b]	N[b]	Y[a]
Sheehan et al., 2012 [34]	Y[a]	N[b]	Y[a]	Y[a]
Ojeleye et al., 2013 [35]	Y[a]	N[b]	Y[a]	Y[a]
Walsh et al., 2013 [36]	Y[a]	N[b]	Y[a]	Y[a]
Tenorio et al., 2013 [37]	Y[a]	N[b]	Y[a]	Y[a]
Moja et al., 2014 [38]	Y[a]	N[b]	Y[a]	Y[a]
Nguyen et al., 2014 [39]	Y[a]	N[b]	Y[a]	Y[a]
Fiander et al., 2015 [40]	Y[a]	N[b]	Y[a]	Y[a]
Pan American Health Organization, 2016 [6]	N[b]	Y[a]	N[b]	Y[a]

[a]Y: yes
[b]N: no

References

. Pan American Health Organization. Webcitation. Pan American Health Organization; 2016. eHealth in Latin Americathe Caribbean: interoperability standards review [accessed 2017-07-14]http://www.webcitation.org/6rwrfeqBJ[Webcite Cache]
. Police RL, Foster T, Wong KS. Adoption and use of health information technology in physician practice organisations: systematic review. Inform Prim Care 2010; 18(4):245-58[FREE Full text][Medline]
. Main C, Moxham T, Wyatt J, Kay J, Anderson R, Stein K. Computerised decision support systems in order communication for diagnostic, screening or monitoring test ordering: systematic reviews of the effects and cost-effectiveness of systems. Health Technol Assess 2017 Jul 14; 14(48):1-227[FREE Full text][Medline][CrossRef]
0. Chaudhry B, Wang J, Wu S, Maglione M, Mojica W, Roth E, Morton SC, Shekelle PG. Systematic review: impact of health

information technology on quality, efficiency, and costs of medical care. Ann Intern Med 2006 May 16; 144(10):742-52[Medline]
. Jamal A, McKenzie K, Clark M. The impact of health information technology on the quality of medical and health care: a systematic review. HIM J 2009; 38(3):26-37[Medline]
. Wollersheim D, Sari A, Rahayu W. Archetype-based electronic health records: a literature review and evaluation of their applicability to health data interoperability and access. HIM J 2009; 38(2):7-17[Medline]
. Chan AJ, Chan J, Cafazzo JA, Rossos PG, Tripp T, Shojania K, Khan T, Easty AC. Order sets in health care: a systematic review of their effects. Int J Technol Assess Health Care 2012 Jul; 28(3):235-40[Medline][CrossRef]
. Sheehan J, Sherman KA. Computerised decision aids: a systematic review of their effectiveness in facilitating high-quality decision-making in various health-related contexts. Patient Educ Couns 2012 Jul; 88(1):69-86[Medline][CrossRef]
. Ojeleye O, Avery A, Gupta V, Boyd M. The evidence for the effectiveness of safety alerts in electronic patient medication record systems at the point of pharmacy order entry: a systematic review. BMC Med Inform Decis Mak 2013 Jul 01; 13:69[FREE Full text][Medline][CrossRef]
. Walsh C, Siegler EL, Cheston E, O'Donnell H, Collins S, Stein D, Vawdrey DK, Stetson PD, Informatics Intervention Research Collaboration (I2RC). Provider-to-provider electronic communication in the era of meaningful use: a review of the evidence. J Hosp Med 2013 Oct; 8(10):589-97[FREE Full text][Medline][CrossRef]
. Tenório J, Cohrs F, Pisa I, de Fátima Marin H. Personal health record: a review on current models and experiments. J Health Inform 2013; :91-97[FREE Full text]
. Moja L, Kwag KH, Lytras T, Bertizzolo L, Brandt L, Pecoraro V, Rigon G, Vaona A, Ruggiero F, Mangia M, Iorio A, Kunnamo I, Bonovas S. Effectiveness of computerized decision support systems linked to electronic health records: a systematic review and meta-analysis. Am J Public Health 2014 Dec; 104(12):e12-22[Medline][CrossRef]
. Nguyen L, Bellucci E, Nguyen LT. Electronic health records implementation: an evaluation of information system impact and contingency factors. Int J Med Inform 2014 Nov; 83(11):779-96[Medline][CrossRef]
0. Fiander M, McGowan J, Grad R, Pluye P, Hannes K, Labrecque M, Roberts NW, Salzwedel DM, Welch V, Tugwell P. Interventions to increase the use of electronic health information by healthcare practitioners to improve clinical practice and patient outcomes. Cochrane Database Syst Rev 2015 Mar 14; (3):CD004749[Medline][CrossRef]
